# Does sharing the electronic health record in the consultation enhance patient involvement? A mixed‐methods study using multichannel video recording and in‐depth interviews in primary care

**DOI:** 10.1111/hex.12320

**Published:** 2014-12-18

**Authors:** Heather Milne, Guro Huby, Susan Buckingham, James Hayward, Aziz Sheikh, Kathrin Cresswell, Hilary Pinnock

**Affiliations:** ^1^ eHealth Research GroupCentre for Population Health SciencesThe University of EdinburghEdinburghUK; ^2^ Faculty of Health and Social StudiesUniversity College Østfold and School of Health in Social ScienceEdinburghUK; ^3^ Centre for Population Health SciencesThe University of EdinburghEdinburghUK; ^4^ Allergy and Respiratory GroupCentre for Population Health SciencesUniversity of EdinburghUK

**Keywords:** consultation skills, electronic health record, information technology, patient involvement, primary care

## Abstract

**Background:**

Sharing the electronic health‐care record (EHR) during consultations has the potential to facilitate patient involvement in their health care, but research about this practice is limited.

**Methods:**

We used multichannel video recordings to identify examples and examine the practice of screen‐sharing within 114 primary care consultations. A subset of 16 consultations was viewed by the general practitioner and/or patient in 26 reflexive interviews. Screen‐sharing emerged as a significant theme and was explored further in seven additional patient interviews. Final analysis involved refining themes from interviews and observation of videos to understand how screen‐sharing occurred, and its significance to patients and professionals.

**Results:**

Eighteen (16%) of 114 videoed consultations involved instances of screen‐sharing. Screen‐sharing occurred in six of the subset of 16 consultations with interviews and was a significant theme in 19 of 26 interviews. The screen was shared in three ways: ‘convincing’ the patient of a diagnosis or treatment; ‘translating’ between medical and lay understandings of disease/medication; and by patients ‘verifying’ the accuracy of the EHR. However, patients and most GPs perceived the screen as the doctor's domain, not to be routinely viewed by the patient.

**Conclusions:**

Screen‐sharing can facilitate patient involvement in the consultation, depending on the way in which sharing comes about, but the perception that the record belongs to the doctor is a barrier. To exploit the potential of sharing the screen to promote patient involvement, there is a need to reconceptualise and redesign the EHR.

## Introduction

Medical consultations can be considered as the meeting of two ways of understanding and managing ill health.^1^, ^2^ Mishler suggested that within the consultation, the patient's experiential narrative of illness (the ‘voice of the life world’) meets, and is subsumed by, the medical conception of illness as a disease, defined and classified by medical science (the voice of medicine).^1^, ^3^ Analysing the role of the medical record in the 1990s, Berg argued that reading and writing the record is core to the process of diagnosis through which the doctor constructs a ‘patient’ with a ‘manageable problem’.^4^ According to Timmermans and Berg^5^ the record:

Does not merely represent but actively *mediates* the clinical encounter by directing the clinician's gaze. Note‐taking and reading medical records transforms information by assembling it in specified formats.^5^

So the record can be understood to facilitate doctors’ systematic channelling of patients’ experiential narratives into a medically defined disease/diagnosis. In addition, others have argued that the patient–doctor interaction during the consultation can be shaped by the doctor's use of the record to indicate turn taking and to signal the conclusion of the consultation.^6^ Hence, the record, as traditionally used, plays an important role within the consultation and supports a balance of power in favour of the doctor and the medical interpretation of the illness.^1^, ^7^

Over the past 30 years, the patient's role as an essential partner in their own health care has been increasingly recognized and patient involvement in consultations has become the accepted ideal in medical practice.^8^, ^9^ This involves a shift along a scale of involvement outlined by Thompson from paternalism towards shared decision making, and informed decision making (see Table 1 for definitions).^10^ However, exactly how patient involvement should be defined and what it involves remains a subject of debate.^9^, ^10^, ^11^, ^12^, ^13^, ^14^, ^15^, ^16^, ^17^, ^18^, ^19^, ^20^, ^21^, ^22^, ^23^

**Table 1:** Thompson's taxonomy of involvement^10^

Paternalism, where the professional knows best and patient involvement is limited to being given information or giving consent Professional‐as‐agent, where professionals possess the technical expertise, but patient preferences are incorporated into their decision making Shared decision making, where both the process and outcome of decisions about treatment options are shared between patient and professional Informed decision making, where the technical expertise is transferred to the patient, who makes the final decision.

Shared decision making, patients and clinicians deciding together on treatment, is the dominant ideal in much patient involvement research.^17^, ^18^, ^19^, ^20^ Most studies of this approach tend to strive ‘towards the systematization of practices of involvement’ to model and measure ‘competencies’.^20^ Donetto and Cribb^20^ argue this has led to the medicalization of involvement by leaving the definition to professionals and policy makers rather than patients. Shared decision making may also under‐represent patients’ desire to be involved and their perceptions of what this means, both of which vary depending on multiple contextual factors.^10^, ^11^, ^12^, ^15^, ^20^, ^21^, ^22^, ^23^

In contrast, research starting from the perspective of the patient has found their understandings of involvement tend to focus on communication. This includes a two‐way sharing of information, and feeling supported in making contributions to the discussion of choices, but not always being directly involved in making decisions.^10^, ^11^, ^12^, ^13^, ^14^, ^24^ In this study, we followed this second strand of research into patient involvement and adopted an inductive approach.^11^ Patients defined involvement themselves and discussed whether sharing the screen assisted this.

It has been suggested that having access to their electronic health‐care record (EHR) may support patients’ involvement by giving them access to more information about their care.^25^, ^26^, ^27^ However, patients do not routinely use the health‐care record for their own information purposes, and tools accessed outside the consultation, such as electronic patient portals, seem to have little effect on patient involvement.^28^, ^29^, ^30^ This may be because patients feel that accessing their health‐care record is of limited value unless a health‐care professional is present to discuss the salience of what they are reading.^15^, ^18^, ^19^ Facilitating patient involvement by sharing the EHR within the consultation is rarely explored.^17^

Sharing the screen is a recognized strategy described in the etiquette of using computers in GP consultations,^26^ and one study has noted sharing to occur in 8% of GP consultations.^31^ Previous observational research has suggested that sharing the computer screen and information from the EHR during consultations may facilitate communication, which in turn may support involvement.^32^, ^33^ During tests of a prototype‐shared touch screen, older patients considered that viewing charts and images from their medical records ‘enhanced communication with their doctors and aided understanding’.^34^ Other studies have suggested that visual presentations of information may support patient recall and communication of risk.^35^, ^36^ Given the dominance of the visual within Western culture, which closely associates seeing with knowing and understanding,^37^ using the screen as a prop for discussion is potentially a powerful facilitator of patient involvement.

A recent observational study of GP consultations defined patients as adopting either a ‘dyadic’ or ‘triadic’ approach to the computer, excluding or including it respectively, by adopting ‘screen‐watching’ and ‘screen‐controlling’ behaviours, which could be facilitated by the orientation of the screen on the desk.^38^, ^39^ Pearce *et al*.^39^ argue that patients adopting a ‘triadic’ approach use the computer to direct the flow of the conversation, and so shift the traditional balance of power within the consultation. They suggest that by invoking information presented on the screen, patients accrue power and may use this to challenge the clinician.^39^

However, the above observational research does not explore patients’ and professionals’ own interpretations of their behaviour, and provides little insight into experiences of screen‐sharing in relation to patient involvement.^30^, ^31^, ^32^, ^33^, ^39^ In this study, we sought to address these issues by exploring how screen‐sharing is accomplished, and whether it is perceived to support patient involvement within the consultation.

## Methods

This study presents an analysis of a subset of video recordings and interviews with patients and GPs collected as part of a larger project, INTERACT‐IT, which studied the use of information technology (IT) across a variety of health settings including accident and emergency, oncology clinics and primary care. Full details of the methodology are in the final report.^40^ The study was conducted in the UK between 2009 and 2011, with ethical approval from the Leeds East Multicentre Research Ethics Committee (MREC: 09/H1306/60) and governance approval from all participating NHS Trusts.

### Practice and participant recruitment

We purposively sampled four primary care practices to ensure a spread of demography, size and IT systems and invited up to three GPs in each practice to participate. Patients were advised about the research when booking an appointment for a recorded clinic. When they arrived for their consultation, a researcher discussed the study with them, and invited them to participate. GPs and patients were invited to consent to (i) filming the consultation; (ii) allowing the researchers to view and analyse the film; and (iii) being approached for a post‐consultation interview. Further consent to be audio recorded was taken prior to the post‐consultation interviews.

We recruited nine GPs across the four sites, given the pseudonyms ‘Hills’, ‘Church’, ‘Seaside’ and ‘House’ to maintain anonymity. From 119 patients who agreed to be videoed, 116 consented to their consultations being analysed. The quality of two videos was too poor for analysis, so in total 114 consultation videos were reviewed.

### Multichannel video recording

Observational data were collected by multichannel video recording using three discreetly located cameras in the consultation room.^38^ We recorded one or two whole clinics for each participating GP (excluding consultations for which the patient declined consent).

We then selected video‐recorded consultations for paired, but separate, in‐depth interviews with the GP and patient about their experience of the encounter and the role of IT within it. We aimed for one or more paired interviews from each recorded clinic, relating to consultations in which IT had featured (but not necessarily cases where the screen had been shared), and for which patients consented to be contacted and were available to take part in interviews within a week of the consultation. All interviews were conducted by HM or the senior project researcher (FT).

### Interview data collection and analysis

Data collection and analysis was a three‐stage iterative process triggered by a finding that ‘sharing the screen’ was a key theme emerging from the analysis of both GP and patient post‐consultation interviews.

GPs and patients from selected consultations separately participated in in‐depth post consultation interviews.^41^, ^42^ In line with principles derived from visual anthropology, these interviews were designed to gain an insight into participants’ reflections and interpretations of what was presented in the visual media.^43^, ^44^ They explored people's expectations of GP consultations, and what they liked, disliked and would change about the use of IT within them. Participants viewed the wide‐angled video recording of their consultation and provided a commentary on this, particularly reflecting on the use of IT. We collected 10 sets of interviews with both patient and GP from the same consultations. In a few cases, this was not possible (for reasons of ill health or other commitments). All GPs provided at least one interview, but for three consultations the interview was provided by the patient only, and in three by the GP only. In total, 13 GP and 13 patient post‐consultation interviews (from nine GPs and 13 patients) were conducted (26 in total).

Consultation videos with interviews were transcribed verbatim and organized into worksheets (by HM and SB) in which the conversation, observations of patient/professional actions, use of the computer and the commentary from the post‐consultation interview were combined (see Table 2). NVivo 9 [QSR International, Melbourne, Vic., Australia] was used to store and organize data during worksheet construction and on‐going thematic analysis.

**Table 2:** Construction of worksheets

Time span	Content	Health professional movement	Patient movement	Screen capture	Health professional commentary	Patient commentary
1 : 10–1 : 13	Transcriptionof the verbal dialogue between health‐care professional and patient	Selected observed physical actions of the health‐care professional	Selected observed physical actions of the patient	Observation of computer screen display	Health‐care professional's commentary on the consultation	Patient's commentary on the consultation

Worksheets were used as an analytical tool in conjunction with the videos and the interview transcripts to identify aspects of the consultations that patients and/or GPs considered significant. Screen‐sharing occurred in only six of the consultation/interview sets, but was nevertheless identified as a significant theme: 13 of 20 interviewees discussed screen‐sharing. Discussion within the multidisciplinary team led to consensus that screen‐sharing was a theme warranting further exploration.

Seven semi‐structured telephone interviews with additional patients explored their perceptions of involvement and the emergent theme of screen‐sharing. The interviews focused on these two topics and were not prompted by watching the videoed consultation. These patients had indicated their willingness to participate in interviews and were available within the study time frame, but they were not selected on the basis of having shared the screen. The interviews were transcribed verbatim and used to inform the on‐going thematic analysis of screen‐sharing following a constant comparative approach.^45^, ^46^, ^47^, ^48^, ^49^ Interviews ranged in length from 15 to 90 min, with the semi‐structured telephone interviews generally being shorter than the interviews involving a video commentary.

Finally, all the consultation videos were reviewed to assess screen‐sharing frequency and to further our understanding of how sharing comes about in practice and the significance of this to patients and professionals. This informed the thematic analysis, which triangulated data from all three stages of the data collection process. Characteristics of the consultations and all participants interviewed are given in Table 3.

**Table 3:** Characteristics of participants

General Practice ID	GP ID Sex	Consultations analysed	Consultations with screen‐sharing	Consultations in which patient looked at the screen	Interviewed patient ID	Patient Sex and age
Church	GP1 Male	7	0	4	ChurchGP1P2	Female48
	GP2 Male	7	2	1	ChurchGP2P8	Female 72
	GP3 Male	6	1	0	–	
Hills	GP1 Male	17	4	8	HillsGP1P9	Male 61
					HillsGP1fP1	Male 52
					HillsGP1P7	Female 67
	GP2 Female	25	4	6	HillsGP2P6	Female 36
					HillsGP2P9	Female 81
					HillsGP2fP7	Female 51
Seaside	GP1 Female	28	5	3	SeasideGP1P4	Female 82
					SeasideGP1fP16	Female 52
					SeasideGP1fP4	Female 78
					SeasideGP1fP5	Female 72
					SeasideGP1fP13	Male 68
	GP2 Male	17	2	3	SeasideGP2P5	Female 81
					SeasideGP2P7	Female 79
					SeasideGP2fP5	Male 51
					SeasideGP2fP2	Male 54
					SeasideGP2fP3	Female 63
					SeasideGP2fP6	Female 82
	GP3 Male	3	0	0	–	
House	GP1 Female	4	0	1	HouseGP1P4^a^	Male 57
TOTAL		114	18	26	13 with commentaries	
					7 additional interviews	

a This patient was filmed in a nurse consultation but at interview chose to discuss his consultations with his GP in detail, and these data are used here.

In the table, above consultations were judged to involve screen‐sharing when GP and patient looked at the screen together and referred to it as part of their conversation, or it was described as screen‐sharing by either party at interview. There were also cases where the patient looked towards the screen but did not verbally refer to it making it difficult to tell as an external observer whether screen‐sharing had taken place. These were classed as the patient looking at the screen rather than as sharing.

## Results

### Patients’ perceptions of involvement

At interview, patients were asked to outline factors which supported their ‘involvement’ in their consultations. The aspects of the consultation identified reflected previous research findings^10^, ^11^, ^12^, ^13^, ^14^, ^24^ and therefore are not discussed in detail. These included feeling listened to; openness and honesty from the GP; receiving information about and discussing illness management choices; and feeling that their opinion was respected. Some patients considered sharing the screen to facilitate their involvement by supporting these factors, while others felt that it was superfluous to the core interaction between them and their doctor.

### How the screen was shared

In 18 (16%) of 114 videoed consultations, GPs and patients were observed sharing the screen, meaning they viewed the EHR displayed on the screen at the same time and referred to it as part of their consultation. (In a further 26 consultations, patients looked at the screen but did not directly refer to this in their conversation with the GP). Viewing the EHR was the most common reason for sharing the screen, but some other resources were also viewed, for example online risk assessment tools and some patient information sheets.

We identified three ways in which the EHR came to be shared. GPs initiated the use of the screen for what we termed ‘convincing’. Both GPs and patients initiated sharing for ‘translating’, and more rarely, patients initiated sharing when ‘verifying’ the accuracy of the record. Two themes emerged as factors influencing screen‐sharing: ‘ownership and confidentiality of the EHR within the consultation’ and ‘design and accessibility of the EHR’.

#### Convincing

The screen enabled GPs to show patients the results of clinical investigations and to support recommendations visually or provide information/education about the biomedical interpretation of their illness:

You can show patients scanned‐in letters, you can show patients information from different websites (Church GP1)

In Example 1 (Table 4) Seaside GP1 shared a graph of the change in kidney function over time with a patient. The screen was being used by the GP as what she termed, ‘a nice visual aid’ to illustrate, educate and convince the patient in support of her biomedical line of reasoning for a treatment:

**Table 4:** Example 1: ‘Convincing’ The GP uses the screen to ‘convince’ the patient. [Seaside GP1P4]

The GP turned the conversation to address patient 4's diabetes management:
**GP**: the other thing we talked about was metformin that was why…
**Patient**: that's right, yes
**GP**: … you were going to come back wasn't it? [*GP turns her head to gesture to screen but remains facing patient*] I had a good look through [and there was] a definite drop in kidney function when you were really ill [*GP points at the screen*]… but the guidelines for metformin are very clear [*GP turns to look at the screen with her hand on the mouse but glances back to the patient twice as she continues to speak*] that it's fine so long as the kidney function is above a level of 30 …
**Patient:** Mmm
*‐ Graph of blood test results is displayed on the computer screen ‐*
**GP**: …and yours is actually officially normal at 62 [*GP glances back at the patient*]
**Patient:** Oh [*patient follows GPs glance and looks at the screen*]
**GP**: It did‐ the lowest it's ever been if we look [*patient looks at the screen and nods*] on that graph [*GP points to screen and glances to patient and back*] is 52 [*GP turns head to face patient finger still on the screen*]
**Patient:** mmm
**GP**: and metformin's fine above 30 obviously monitoring
**Patient:** yes
**GP**: keeping an eye on it [*GP Turns back to screen and closes the graph then turns to face patient*] *[patient follows GPs gaze to the screen and back]*…. I think personally we should start you on a low dose of Metformin.

[What is] really good is you can do graphs so it shows you how the values have changed over time … I think is really helpful because … you want to try and educate them about the disease process. (Hills GP2)

In this context, the screen was being used as a tool to help ‘patients understand their results’ and ‘buy into treatment’ (Hills GP2), that is to convince them of the translation of their illness into the ‘voice of medicine’.^1^

#### Translating

Translating could be initiated either by the GP or by the patient using the information on the screen to translate between the medical and life‐world meanings of illness and disease. This was done to further the understanding of either the GP (Example 2) or the patient (Example 3).

In Example 2 (Table 5), the GP initiated use of the screen to facilitate translation between the patient's requirement for a supply of tablets and the list of medicines in the EHR. The GP commented ‘showing [patients] something on the computer … is another way of communicating’ (Church GP2). In a two‐way process, viewing the written word on the screen aided the patient's recognition of the word printed on the box of tablets potentially overcoming misunderstandings due to mispronunciation of complex pharmacological terminology. Additional clarification for the GP was drawn from the patient's experience of taking the tablet (for example confirming dosage frequency or the ‘chewy’ nature of the tablet).

**Table 5:** Example 2: ‘Translating’ GP uses the screen to ‘translate’ the repeat medication. [ChurchGP2P8]

As GP and patient discussed which repeat medications needed prescribing, the following interaction using the screen occurred:
**GP:** If I just look at your list here, you've got your quinine *(GP Looks at screen and turns it to face patient; left thumb on edge of screen)*
**Patient:** Yes *(patient leans forward to look at screen)*
As the discussion continued when the GP named a tablet she did not recognize she leant forward to try and see the screen again, as if hoping that seeing the name written would prompt her memory.
**GP:** Okay…and you need some bisoprolol? as well do you? *(Points with left finger on screen)*
**Patient:** Which one's that one? *(Leans forward to see screen)*
**GP:** That's your beta blocker that you take twice a day *(Points with left finger on screen; glances at patient)*
**Patient:** Yes, I need that. That's the chewy one?
**GP:** No, there's the chewy one
**Patient:** No, I don't need that one then. *(Sits back)*

Similarly, the screen could be used to support translation in the opposite direction, from medical terminologies and interpretations into a form that was meaningful to patients, Hills GP1 explained:

I do like the ability to have the patient interacting with the computer … I want to use it as part of the consultation… [so] I can share with the patient and identify any areas that need further explanation (Hills GP1)

Patients also initiated viewing of the screen to aid translation by actively pursuing an explanation of what they saw, as exemplified by Example 3 (Table 6). It is notable that the patient, not the doctor, read blood test results from the screen enabling them to actively control the explanation they received about the medical information displayed. For example, a patient could prompt ‘translation’ of anomalous results between medical/statistical definitions of ‘abnormal’ and the relevance to their well‐being as someone else explained:

**Table 6:** Example 3: ‘Translating’ The patient uses the screen to ‘translate’ their blood test results. [HillsGP1P8]

In this consultation, the patient responded to the GP's opening question ‘how can I help you today?’ by stating ‘I was in a few weeks ago’ and immediately pointed at the screen which was angled towards him.
This prompted the GP to turn to the screen and, using the mouse, retrieve the pathology report saying ‘Yes, for your blood tests’. As the doctor did this, the patient leaned towards the screen and read out aloud:
**Patient:** Oh ‘abnormal’.
**GP:** ‘Yes, your cholesterol is up. Higher than it should be. So that's what that one's about. We need to talk about that.’

Results out of the normal range are highlighted very effectively … in red … looking at it together she's explaining the things she thinks are important but it also gives me the option to say ‘well what about that one there’. I'm not just sat there passive the other side of the desk. (House GP1P4)

#### Verifying

In addition to requesting explanations, being able to see the screen enabled patients to question the accuracy and veracity of their EHR, as in Example 4 (Table 7).

**Table 7:** Example 4: ‘Verifying’ The patient uses the screen to ‘verify’ their EHR. [Hills GP1P4]

This patient's appointment focused on the problems she was having with her thyroid and her knee. However, in the closing phases of the consultation, the patient pointed to her record displayed on the screen, which was turned slightly towards her, to ask a question:
**Patient:** Just looking on there. Being nosy. But *[patient points at the screen]* a healed leg ulcer?
**GP:** Oh?
**Patient:** I'll tell you why I'm asking because my Mam, used to have… two very bad leg ulcers, and I've got like a red mark… which I'm always frightened that it could turn into something… Is that what that [is about]?
**GP:** No. That's just the name of the form that the nurses have used to check the blood pressure in your leg. We sometimes use that for leg ulcers to see what the circulation is like.
**Patient:** Oh. I'm being hypersensitive.
**GP:** No, no don't worry. They're your notes so feel free to ask anything about what's on them. They're your notes they're not really my notes.
**Patient: ** *OK (laughter)*

From the conversation in Example 4, it seemed that the patient had observed an unexpected reference to leg ulcers in her EHR. Her question implied both concern about an inaccuracy in her medical history and also anxiety about the implications whether the entry alluding to leg ulcers was actually accurate.

Although all the GPs were seen to share the screen at times, only Hills GP1 was explicit that this was a basic right for patients because ‘it's information about them, and it's information that they should know about and understand, and be able to query as well, or correct’. Most considered that the EHR and the screen that displayed it belonged to them rather than to the patient. As Church GP3 put it, ‘I do use [the screen] for them… although it's mainly mine’.

Several patients talked about being able to see the screen as increasing a sense of openness, which has previously been identified as a key aspect of enabling patient involvement and trust:^13^

I don't think they're doing anything to hide anything from you, they're not turning the screen away so you can't look at it whereas in years gone by… you wouldn't get to see what they were writing… With the computer… you're kept in the loop basically. (Hills GP1P11)

These results outline a range of ways in which the screen was shared, and identify three ways in which patients felt the screen facilitated involvement: First being able to see it could create a sense of openness and inclusion; second, the visual nature of the information displayed on the screen facilitated questions about medical test results; finally, it made it possible to question the record's content. However, interviews carried out with both patients and GPs suggested that using the screen to facilitate involvement was influenced by understandings of the ‘ownership’ of the EHR and the accessibility of the information displayed.

### Ownership and confidentiality of the EHR within the consultation

Most patients presented the screen as belonging to the GP rather than as something that they could, and should, view. Many said that they would like to read what was written on their notes, but described this as just ‘being nosy’ (HillsP37). Several went further saying they felt they ‘shouldn't read them’ because the computer and screen was not theirs to read.

[It's] his computer, his screen… I feel a bit nosy when I'm looking… I'd like to look at my notes… but you feel it's personal to the doctor, this is the thing isn't it. (Seaside GP2fP5)

Patients’ lack of confidence in their right to read the screen reflects the currently accepted dynamics of medical practice and may have been influenced by their own and the GPs’ perception that the GPs owned the screen and (by implication) the EHR. Only one GP explicitly encouraged the patient to consider the EHR as belonging to them commenting ‘they're your notes; they're not really my notes’.

Only one patient expressed an active desire not to see her notes because, she said, ‘if I was dying I wouldn't want to know’ (HillsGP2P9). It was unclear whether this was because she was afraid of what she might see without the opportunity for an explanation or discussion, or whether it was because she preferred her GP to keep certain things from her.

Some patients explained that viewing the screen was unnecessary because the GP ‘just tells me what's on it and I take their word for it’ (Seaside GP2fP2). They trusted that their GP would ‘give you the information you need’ (HillsGP2P6), and so they did not need to see the screen for themselves. Several people mentioned that the screen was not necessarily an important part of the interaction because they were there to talk to the GP, and some hinted that looking at it could even imply a lack of faith in the GP rather than trusting that ‘the doctor knows what they're doing’ (SeasideGP1P4). Viewing the screen was not an expected or necessary part of the consultation because the key desired interaction was to talk with the GP. For example, SeasideGP2P5 remarked that she was ‘just too busy chatting to the doctor’ to look at the screen.

**Figure 1:**
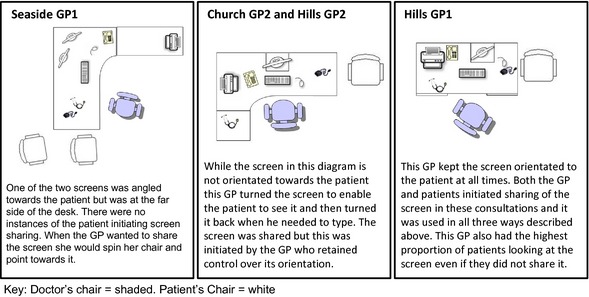
Examples of the consulting room layouts.

Finally, patients may bring a companion into consultations with them and some GPs were concerned that sharing the screen in these situations risked breaking confidentiality (Fig. 1). For example, while one GP explained that he liked to ‘turn the screen and show them results’, he also often turned the screen away because ‘you don't want anybody [accompanying the patient] to be able to look over your shoulder’ (Church GP1). A few patients were concerned that having the screen displayed all the time might accidentally lead to breaches of confidentiality:

It would be a problem if they'd gone into someone else's file by mistake. So I think it's a good idea that you can't see it. (Hills GP2P6)

Patients generally perceived the EHR on the screen as the domain of the doctor, potentially limiting the screen as a facilitator of communication and involvement. The positioning of the screen within the consulting space could reinforce this perception, for example if the screen was only visible when the GP chose to turn it towards them.

### Design and accessibility of the EHR

The design of the EHR in terms of what and how information was displayed, and where the screen was placed on the desk may have influenced perceptions of who should use the EHR and how. Figure 1 illustrates the variation in the positioning and deployment of the screen by the GPs in the study.

Both GPs and patients expressed concerns that the information presented on the EHR ‘isn't always patient friendly’ (Hills GP2). However, only Hills GP1 talked about deliberately positioning the screen for patients to be able to see it:

[I] keep the computer at an angle such that it's fully visible to them and can be part of the consultation as well. (Hills GP1)

Other GPs explicitly turned the screen towards the patient (Church GP2 and Hills GP2), or swivelled their chair and pointed to the screen (Seaside GP1) to signal sharing of the screen. This strategy left the screen in the control of the GP who could invite sharing (and close that component of the consultation) at their discretion.

However, these efforts may be wasted if the small font size and complex layout of the information meant that patients could not always see what they were being shown:

[The GP will] turn it and say ‘you see what it says here that – such‐and‐such?’ so I think ‘well, it doesn't matter anyway because I can't see the… thing! (Hills GP2fP7)

Some patients who felt that sharing the screen facilitated their involvement in the consultation and meant they were not just sitting ‘passive the other side of the desk’ (HouseGP1P4) suggested that the design of the screen could be modified to support their involvement. They suggested using ‘fancy graphics’ that ‘could show a visualization of what's happening’ (HouseGP1P4) and having a bigger display ‘so when you're sitting the other side [of the desk] you can see what's going up on the screen’ (HillsGP1P9), or having two screens with one angled towards the patient.

Currently, the software which provides access to the EHR is designed for the sole use of the health professional. None of the available systems consider or accommodate the possibility that patients may share the screen. This may in turn reinforce both GP and patient perceptions that the record is exclusively the GP's domain, acting as a further barrier to sharing.

## Discussion

Our data augment understandings about how the EHR on the screen is shared and its nuanced role within the consultation. Sharing the screen has the potential to enhance aspects of involvement, such as two‐way communication and a sense of openness, by facilitating translation or enabling patients to question the veracity of the EHR. However, when used by the clinician to convince the patient of the medical model, it may paradoxically reduce involvement. Perceptions that the EHR is the doctor's domain, and a screen design that privileges doctors’ understanding of the content over that of patients, limit the use of the screen to facilitate involvement.

Berg understood the process of reading and writing the health‐care record as central to the doctor's practice of systematically channelling the patient's concerns into a ‘manageable problem’ which could be treated clinically.^4^ From this perspective, the record facilitates the dominance of the medical interpretation of the patient's illness and supports the power imbalance between doctor and patient within the consultation. There have been attempts recently to redress this power imbalance by rendering the record more accessible to patients outside the consultation. However, low uptake rates^30^, ^50^ and disparities in Internet access to Web‐based patient portals suggest that sharing the record within the consultation may have greater potential for facilitating involvement. As suggested elsewhere, our analysis suggests the EHR viewed on the screen and discussed within the consultation has the potential to facilitate patient involvement.^27^, ^39^ By embedding sharing of the record within the consultation (as opposed to unsupported access to records), some of the challenges of health literacy may also be addressed through open discussion of patients’ health information.^51^

Moving away from being purely the doctor's tool that Berg described,^4^, ^5^ the EHR may become a tool used by the patient as well as the professional, to facilitate two‐way translations between the ‘voice of the life world’ and the ‘voice of medicine’.^1^ However, our data suggest that this potential depends on how the screen is shared and, importantly, perceptions of ownership of the EHR.

We identified three ways in which screen‐sharing occurred (‘convincing’, ‘translating’ and ‘verifying’) that reflect differing roles for the record in the patient–doctor interaction and differing extents of patient involvement. When using the screen in ‘convincing’, the power of the visual is employed by the GP to ‘convince the patient of the existence of a disease and/or efficacy of treatment. While this provided the patient with information it also leads to the paradox inherent in health education in general that providing information may enable patients to make more informed decisions about their health, but may also subjugate the patient's illness narrative to the biomedical presentation.^52^ The GP's use of the screen to ‘convince’ may limit rather than facilitate patients’ involvement in the consultation.

In contrast, sharing the screen to help ‘translate’ between the ‘voice of the life world’ and the ‘voice of medicine’^1^ uses the EHR to facilitate communication and patient involvement. This may support shared decision making in which the patient and professional discuss information from the EHR, and reach a decision together.^10^ Patient involvement is complex, but creating the opportunity for patients to question (and correct) what is recorded in their record may be a small move towards a more egalitarian relationship by potentially allowing the patient to reassert ownership of the ‘story’ held in their notes.

However, for sharing the screen to support patient involvement, the screen needs to be more accessible to patients both through a change in the way we conceptualise the record, and in its physical position and presentation of information. Practical designs that enable effective sharing, perhaps along the lines of Piper and Hollan's prototype large touch screens are needed.^34^ Including visual images of medications, or animations that can be used in medical explanations, may also be useful. Patients suggested providing larger and/or additional screens that they could view. Redesigning the screen to be shared is challenging as the need remains to protect patient confidentiality and to enable patients to view information in an accessible language while allowing the medical professionals to continue to use concise and specific medical terminologies. Having two screens, one of which is routinely shared, opens up the possibility of using the EHR to support communication while allowing confidentiality to be protected.

However, design adjustments may only facilitate involvement if the way in which the screen is used supports ‘translating’ and ‘verifying’. This will require a change in attitudes to the ownership of the EHR. It is notable in our data that only one GP considered it the patient's right to view the screen: others viewed the computer as their tool, not the patient's. Training for GPs that presents the medical record as belonging to the patient and encourages the practice of sharing the information on the screen may contribute to this shift.

### Strengths and limitations

The salience of screen‐sharing as an important topic meriting further exploration emerged from the larger INTERACT‐IT project,^40^ in which a robust, iterative process of data collection and analysis triangulated data from three different sources and across four different primary care settings. Moreover, constant discussion of themes among the interdisciplinary team and using emergent themes to construct topics and questions for subsequent semi‐structured interviews strengthened this finding. Although the rate of screen‐sharing was twice that in previous studies,^31^ only a small proportion of videoed consultations involved genuine screen‐sharing between doctor and patient, and our number of interviews is small.

Reflecting population use of health‐care services, half of interviewed patients were over 65 years of age and may have been less likely than younger patients to prefer an active role in the consultation^23^ and to be less comfortable with computers. This may have influenced our findings. Reading the screen also assumes literacy among the patients, limiting its use to people with sufficient English literacy skills.

In addition, our data derive from UK primary care consultations, in which the use of the EHR is the norm.^40^ IT systems and the challenges they present are international,^53^ although our findings may not translate directly to settings where the medical record is not fully electronic, or the patient is acutely ill, or where the EHR has limited historical information about the patient. To inform assessment of transferability to other health‐care contexts,^54^ we have provided contextual description about the characteristics of the patients and their GPs (Table 3) and a description of the physical set‐up of the computer on the desk (Figure 1). Other organisational factors within individual practices may also impact on how the EHR is viewed and attitudes to sharing the screen.

## Conclusions

Our data suggest that depending on how the screen is employed it can facilitate patient involvement or further subjugate the ‘voice of the life world’^1^ to that of medicine. This insight could inform consultation skills training. Shared use of the record is, however, limited by the perception that the screen and EHR are the property of the doctor and not for the patient to view. To support involvement, the EHR needs to be reconceptualised as the joint property of patient and doctor. A second challenge lies in designing an EHR which retains the precision of medical language and observations while simultaneously translating the record into modes of presentation which are useful to the patient.

## Source of funding

This work was funded by the NHS CFH Evaluation Programme (NHS CFHEP 010) commissioned by NHS Connecting for Health (NHS CFH) through the Department of Health (DH) Research & Development (R&D) Directorate, United Kingdom. The views expressed in this publication are those of the authors and not necessarily those of the NHS, the NHS CFH Evaluation Programme or the Department of Health. HP was supported by a Primary Care Research Career Award from the Chief Scientist's Office of the Scottish Government at the time of the study. AS was supported by a Harkness Fellowship in Health Care Policy and Practice from The Commonwealth Fund.

## Conflicts of interest declaration

All authors declare they have no conflicts of interest. All authors have completed the Unified Competing Interest form at www.icmje.orge/coi_disclosure.pdf (available on request from the corresponding author).

## Contributorship

HP, AS, KC and GH, conceived the idea for the study, developed the protocol and secured funding; HP was the principal investigator and with GH, AS and KC led study administration, data analysis and interpretation of results. HM and JH undertook data collection, handling of data and data analysis. SB assisted with management of data and analysis. All authors had full access to all the data and were involved in interpretation of the data. HM with HP and GH wrote the initial draft of the study, to which all the authors contributed. HP is the study guarantor.

## Exclusive license

The Corresponding Author has the right to grant on behalf of all authors and does grant on behalf of all authors, a worldwide licence to the Publishers and its licensees in perpetuity, in all forms, formats and media (whether known now or created in the future), to (i) publish, reproduce, distribute, display and store the Contribution; (ii) translate the Contribution into other languages, create adaptations, reprints, include within collections and create summaries, extracts and/or, abstracts of the Contribution; (iii) create any other derivative work(s) based on the Contribution; (iv) to exploit all subsidiary rights in the Contribution; (v) the inclusion of electronic links from the Contribution to third‐party material whereever it may be located; and (vi) license any third party to do any or all of the above.

## Data sharing

We do not have consent to share data.
